# gp100/pmel17 and tyrosinase encode multiple epitopes recognized by Th1-type CD4+T cells

**DOI:** 10.1054/bjoc.2001.2160

**Published:** 2001-11

**Authors:** L S Kierstead, E Ranieri, W Olson, V Brusic, J Sidney, A Sette, Y L Kasamon, C L Slingluff, J M Kirkwood, W J Storkus

**Affiliations:** Departments of Surgery, Medicine, Molecular Genetics and Biochemistry, University of Pittsburgh School of Medicine, Pittsburgh, PA, 15261; Kent Ridge Digital Laboratory, Singapore; Epimmune Corporation, San Diego, CA, 92121; Department of Surgery, University of Virginia, Charlottesville, VA, 22908; University of Pittsburgh Cancer Institute, Pittsburgh, PA, 15261, USA

**Keywords:** tyrosinase, gp 100/pmel17, melanoma, helper T cells, ELISPOT, vaccine

## Abstract

CD4+ T cells modulate the magnitude and durability of CTL responses in vivo, and may serve as effector cells in the tumour microenvironment. In order to identify the tumour epitopes recognized by tumour-reactive human CD4+ T cells, we combined the use of an HLA-DR4/peptide binding algorithm with an IFN-γ ELISPOT assay. Two known and three novel CD4+ T cell epitopes derived from the gp 100/pmel17 and tyrosinase melanocyte-associated antigens were confirmed or identified. Of major interest, we determined that freshly-isolated PBMC frequencies of Th1-type CD4+ T recognizing these peptides are frequently elevated in HLA-DR4+ melanoma patients (but not normal donors) that are currently disease-free as a result of therapeutic intervention. Epitope-specific CD4+ T cells from normal DR4+ donors could be induced, however, after in vitro stimulation with autologous dendritic cell pulsed with antigens (peptides or antigen-positive melanoma lysates) or infected with recombinant vaccinia virus encoding the relevant antigen. Peptide-reactive CD4+ T cells also recognized HLA-DR4+ melanoma cell lines that constitutively express the relevant antigen. Based on these data, these epitopes may serve as potent vaccine components to promote clinically-relevant Th1-type CD4+ T cell effector function in situ. http://www.bjcancer.com © 2001 Cancer Research Campaign


British Journal of Cancer (2001) 85(11), 1738–1745
© 2001 Cancer Research Campaign
doi: 10.1054/ bjoc.2001.2160, available online at http://www.idealibrary.com on IDEAL

### gp100/pmel17 and tyrosinase encode multiple epitopes recognized by Th1-type CD4+T cells

**LS Kierstead[1], E Ranieri[1], W Olson[1], V Brusic[4], J Sidney[5], A Sette[5], YL Kasamon[1], CL Slingluff Jr[6], JM Kirkwood[2,7] and WJ Storkus[1,3,7]**

[1]Departments of Surgery, [2]Medicine, and [3]Molecular Genetics and Biochemistry, University of Pittsburgh School of Medicine, Pittsburgh, PA 15261; [4]Kent Ridge Digital Laboratory, Singapore; [5]Epimmune Corporation, San Diego, CA 92121; [6]Department of Surgery, University of Virginia, Charlottesville, VA 22908; and [7]University of Pittsburgh Cancer Institute, Pittsburgh, PA 15261, USA

**Summary** CD4+ T cells modulate the magnitude and durability of CTL responses in vivo, and may serve as effector cells in the tumour microenvironment. In order to identify the tumour epitopes recognized by tumour-reactive human CD4+ T cells, we combined the use of an HLA-DR4/peptide binding algorithm with an IFN-γ ELISPOT assay. Two known and three novel CD4+ T cell epitopes derived from the gp 100/pmel17 and tyrosinase melanocyte-associated antigens were confirmed or identified. Of major interest, we determined that freshly-isolated PBMC frequencies of Th1-type CD4+ T recognizing these peptides are frequently elevated in HLA-DR4+ melanoma patients (but not normal donors) that are currently disease-free as a result of therapeutic intervention. Epitope-specific CD4+ T cells from normal DR4+ donors could be induced, however, after in vitro stimulation with autologous dendritic cell pulsed with antigens (peptides or antigen-positive melanoma lysates) or infected with recombinant vaccinia virus encoding the relevant antigen. Peptide-reactive CD4+ T cells also recognized HLA-DR4+ melanoma cell lines that constitutively express the relevant antigen. Based on these data, these epitopes may serve as potent vaccine components to promote clinically-relevant Th1-type CD4+ T cell effector function in situ. © 2001 Cancer Research Campaign http://www.bjcancer.com

**Keywords**: tyrosinase; gp 100/pmel17; melanoma; helper T cells; ELISPOT; vaccine

The recent understanding of the molecular basis of T cell recognition of human tumours has allowed for the development of vaccine trials targeting the induction of antigen-specific, tumour-reactive CD8+ CTL. In some cases, CTL reactive against defined tumour epitopes may mediate tumour regression (Kawakami et al, 1998). Many limitations must be overcome in order to achieve optimal efficacy for anti-tumour CTL in vivo, including the identification of means by which to increase antigen-specific CTL numbers, targeting these T cells to the sites of tumour, maintaining T cell viability and function in the potentially immunosuppressive tumour microenvironment, and promoting increased infiltration of tumours by additional immune cells (i.e., NK cells, dendritic cells (DC), macrophages, PMN). Clearly, CD4+ T cell responses may directly impact each of these issues by potentiating both the afferent and efferent aspects of CD8+ T cell function, and by mediating Th1-type DTH reactions within tumour sites, thereby promoting pro-inflammatory cytokine and/or chemokine production (Grohman et al, 1998; Topalian et al, 1996; Bour et al, 1998). Cytokines may enhance locoregional vascular permeability and increase cellular expression of MHC-peptide complexes, thus, improving antitumour T cell efficacy in situ. Chemokines (i.e. IFN-γ dependent IP-10 and Mig) may facilitate lymphocyte infiltration and promote the involution of the tumour-associated neovascular bed (Tannebaum et al, 1998; Sgadari et al, 1996). Furthermore, CD4+ T cells may mediate direct antitumour immunity (Ossendorp et al, 1998) and Th1-type CD4+ T cell responses appear crucial to productive antitumour immune responses (Bour et al, 1998; Aruga et al, 1997; Halliday et al, 1995).

Despite the importance of antitumour CD4+ T cells, tumour antigen-derived peptides that serve as CD4+ T cell epitopes have rarely been defined (Topalian et al, 1996; Storkus and Zarour, 2000). In the present study, we have focused our attention on CD4+ T cell responses restricted by HLA-DR4, in part due to the large information base for this prototype MHC class II allele and its associated peptide-binding preference, as well as, the potential increased risk factor for DR4+ individuals to develop certain forms of cancer (i.e. melanoma, gastric carcinoma, basal cell carcinoma (Barger et al, 1982); Ogoshi et al, 1994; Czarnecki et al, 1993). We have identified novel peptide sequences derived from the gp100/pmel17 and tyrosinase melanoma-associated antigens that elicit IFN-γ production from CD4+ Th1-type T cells obtained directly from the PBMC of HLA-DR4+ melanoma patients and from cultured CD4+ T cells derived from normal HLA-DR4+ donors stimulated in vitro with dendritic cell-based ‘vaccines’. We anticipate that constitutive or induced CD4+ T cell responses directed against these epitopes in HLA-DR4+ melanoma patients may preclude recurrence or mediate the regression of disease, respectively.

### MATERIALS AND METHODS

### Cell lines and media

The T2.DR4 (DRB1*0401+) cell line (kindly provided by Dr Janice Blum, Indiana University School of Medicine, Indianapolis, IN) was used as antigen-presenting cell line in these

*Received 25 June 2001*
*Revised 17 September 2001*
*Accepted 20 September 2001*

*Correspondence to:* WJ Storkus studies. This cell line uniformly expresses HLA-DR*0401 molecules that contain moderate-to-low affinity binding peptides derived mainly from intracellular invariant chain (CLIP, class II-associated invariant chain peptide) due to a genetic deficiency in HLA-DM (Turvy and Blum, 1998). Melanoma cell lines (Mel 526 (HLA-DR4-negative, gp100/pmel17+, tyrosinase+), MEL-SLM2 (HLA-DR4+, gp100/pmel17+, tyrosinase+), M21 (HLA-DR4-dim+, gp100/pmel17+, tyrosinase+), COLO38 (HLA-DR4+, gp100/pmel17+, tyrosinase+) and MEL1011 (HLA-DR4+, gp100/pmel17+/–, tyrosinase+)) were passaged 1:2–1:10 using trypsin-EDTA (GIBCO/Life Technologies, Grand Island, New York) when confluent. All cell lines were maintained in RPMI-1640 supplemented with 10% heat-inactivated fetal bovine serum, 100 IU/ml penicillin, 100 ug/ml streptomycin, and 10 mM L-glutamine (all reagents GIBCO). Mel 526 was the gift of Dr Steven Rosenberg (National Cancer Institute, NCI, Bethesda, MD), MEL1011 was the kind gift of Dr Suzanne Topalian (NCI) and M21 and COLO38 were the kind gifts of Dr Soldano Ferrone (Roswell-Park Cancer Institute, Buffalo, NY). SLM2 was generated at the University of Pittsburgh.

### Peptide selection and synthesis

The protein sequences of the melanoma associated antigens gp 100/pmel17 and tyrosinase were obtained from GENBANK and analysed for HLA-DR*0401 binding peptides using a neural network algorithm (Honeyman et al, 1998). High scoring nine amino acid long sequences were typically extended by three amino acids on either flank using the genomic corresponding sequences. Alternatively, if high scoring nine amino acid long sequences were found to overlap, the longer overlapping sequences were synthesized, with amino acid extensions added to the end(s) of putative DR4-binding sequence(s). Overall, peptides were 15–23 amino acids in length (Table 1) and were synthesized by FMOC chemistry by the University of Pittsburgh Cancer Institute's (UPCI) Peptide Synthesis Facility (Shared Resource). Peptides were >90% pure based on HPLC profile and MS/MS mass spectrometric analysis performed by the UPCI Protein Sequencing Facility (Shared Resource).

### Peptide-binding assay

The peptide-binding determinations were performed using a solid-state competitive-binding assay as previously described (Southwood et al, 1998).

### Isolation of patient and normal donor PBMC-Derived T cells

Forty-to-one hundred ml of patient or normal donor heparinized blood was obtained with informed consent under an IRB-approved protocol and diluted 1:2 with HBSS, applied to ficoll-hypaque gradients (LSM, Organon-Teknika, Durham, NC) per the manufacturer's instructions, and centrifuged at $550 \times g$ for 25 min at RT. Patient and normal donor information is provided in Table 2. Lymphocytes at the buoyant interface were recovered and washed

**Table 1** gp100 and tyrosinase peptides analysed in this study: algorithm scores, solid-state binding $IC_{50}$ values, and summary of CD4+ T cell responsiveness in IFN- γ ELISPOT assays

| | | | Binding affinity ($IC_{50}$) | | T cell responses evaluated in | | |
|---|---|---|---|---|---|---|---|
| **Peptide** | **Sequence** | **Algorithm DRB1*0401 binding score (1st AA position no.)** | **DRB1 *0401** | **DRB1 *0404** | **MEL-NED (of 11)[a]** | **MEL (of 5)[b]** | **ND-IVS (of 10)[c]** |
| gp100/pmel17$_{6-26}$ | KRCLLHLAVIG**ALLAVGATK**V | 4 (15); 5 (9); 6 (12) | – | 335 | **7** | **1** | **7** |
| gp100/pmel17$_{44-59}$** | WNRQLYPEWTEAQRLD | 4 (48) | 136 | 514 | **8** | **1** | **9** |
| gp100/pmel17$_{150-168}$ | VYVW**KTWGQYWQV**LGGPVS | 4 (150); 6 (161) | – | – | 0 | 0 | 0 |
| gp100/pmel17$_{167-189}$ | VSGLSIGTGRAMLGTHTMEVTVY | 3 (170); 4 (178) | – | 372 | 0 | 0 | 0 |
| gp100/pmel17$_{283-304}$ | PGPVTAQVVLQAAIPLTSCGSS | 4 (292); 6 (286) | 2893 | 51 | 0 | 0 | 0 |
| gp100/pmel17$_{377-398}$ | EKVPVSEVMGTTLAEMSTPEAT | 4 (385); 10 (389) | 7014 | 92 | 0 | 0 | 0 |
| gp100/pmel17$_{400-421}$ | MTPAEVSIVVLSGTTAAQVTTT | 3 (400); 4 (407); 5 (408,409) | 148 | 17 | 0 | 0 | 0 |
| gp100/pmel17$_{506-522}$ | EGDAFELTVSCQGGLPK | 6 (510) | 3210 | 3553 | 0 | 0 | 0 |
| gp100/pmel17$_{540-561}$ | LCQPVLPSPACQLVLHQILKGG | 3 (552); 5 (544) | – | 614 | 0 | 0 | 0 |
| gp100$_{599-619}$/pmel17$_{606-626}$ | IVGILLVLMAVVLASLIYRRR | 4 (600,607); 5 (603,604,605) | – | – | 0 | 0 | 0 |
| gp100$_{615-633}$/pmel17$_{622-640}$ | IYRR**RLMKQDFSV**PQLPHS | 4 (615,625); 7 (621) | 51 | 9 | **8** | 0 | **9** |
| tyrosinase$_{56-70}$** | QNILLSNAPLGPQFP | 4 (59); 5 (58) | 5441 | 178 | **7** | 0 | **8** |
| tyrosinase$_{80-95}$ | WPSVFYNRTCQCSGNF | 3 (80,85); 5 (84); 10(83) | – | – | 0 | 0 | 0 |
| tyrosinase$_{156-175}$ | YGQMKNGSTPMFNDINIYDL | 4 (156); 5 (166); 10(159) | 1387 | 953 | **7** | 0 | **6** |
| tyrosinase$_{178-194}$ | WMHYYVSMDALLGGSEI | 6 (181); 8 (182) | NT | NT | **1** | **1** | **3** |
| tyrosinase$_{365-381D}$ | ALHI**YMDGTMSQV**QGSA | 8 (368) | 17 | 31 | **6** | **2** | **9** |
| tyrosinase$_{365-381N}$ | ALHIYMNGTMSQVQGSA | 4 (368) | 1744 | 174 | **3** | **2** | **4** |
| tyrosinase$_{448-462}$** | DYSYLQDSDPDSFQD | 3 (449); 6 (451) | 1684 | – | 0 | 0 | 0 |

[a]Number of HLA-DR4+ melanoma patients (of 11, no evidence of disease (NED) at time of assessment) with freshly- isolated CD4+ T cell reactivity against the indicated peptide as monitored in IFN-γ ELISPOT assays. [b]Number of HLA-DR4+ melanoma patients (of 5, with active disease) with freshly-isolated CD4+ T cell reactivity against the indicated peptide as monitored in IFN- γ ELISPOT assays. [c]Number of normal HLA- DR4+ donors (of 10 evaluated) with CD4+ T cells responding against the indicated peptide in IFN-γ ELISPOT assays after 2 rounds of in vitro stimulation (IVS) with autologous dendritic cells pulsed with peptide. No normal donors displayed significant CD4+ T cell reactivity against any of these peptides prior to in vitro stimulation (see Figure 1C). Patients were considered positive responders if their calculated frequency of peptide-specific spots exceeded the mean + 3 standard deviation values (see Figure 1 legend) obtained from 10 normal HLA-DR4+ donors against that indicated peptide. Known CTL epitopes embedded in these sequences are indicated by shading, while positive T cell responses are in bold and underlined (Kittlesen et al, 1998; Skipper et al, 1996; Ranieri et al, 2000; Brinckerhoff et al, 2000). Peptides indicated with ** represent previously identified HLA-DR4- presented epitopes. Binding data is reported as $IC_{50}$ in nM and represents the concentration of the indicated peptide to block 50% of the binding of a radio-labeled reference peptide to purified HLA-DR4 complexes. IC50 values ≥ 10 000 nM are indicated as–. NT = Not Tested.

*© 2001 Cancer Research Campaign* 

**Table 2** HLA-DR4+ patients evaluated in this study

| Patient | Age | Sex | Stage | Treatment | Status at time of evaluation | DR4 genotype | Tumour antigen expression (RT-PCR) gp100 | tyrosinase |
|---|---|---|---|---|---|---|---|---|
| SLM1 | 71 | F | IV | S, DC/peptide | NED, 1.5 | *0401 | + | + |
| SLM2 | 64 | F | IV | S, IFN-α | NED, 5.0 | *0401 | + | +/– |
| SLM3 | 43 | M | I | S, IFN-α | NED, 3.5 | *0408 | NA | NA |
| SLM4 | 57 | M | IV | S, IFN-α | NED, 3.3 | *0404 | + | + |
| SLM5 | 34 | M | IV | S, IFN-α | NED, 4.1 | *04X | NA | NA |
| SLM6 | 37 | F | I | S, IFN-α | NED, 1.9 | *0401 | NA | NA |
| SLM7 | 46 | M | I | S | NED, 2.3 | *0404 | NA | NA |
| SLM8 | 49 | M | II | S | NED, 4.0 | *0404 | + | + |
| SLM9 | 35 | F | III | S,C,R | NED, 0.3 | *0401 | + | – |
| SLM10 | 74 | M | IV | S | NED, 0.4 | *0401 | NA | NA |
| SLM11 | 58 | M | IV | S | NED, 0.3 | *0404 | NA | NA |
| SLM12[†] | 39 | F | IV | S,C,IFN-α | Stable | *0401 | NA | NA |
| SLM13 | 71 | M | IV | None | Mets, Liver/lung | *0401 | NA | NA |
| SLM14 | 52 | M | IV | C | Mets, Brain | *0408 | NA | NA |
| SLM15 | 66 | M | IV | S, IFN-α | Mets, Liver/lung | *0402 | NA | NA |
| SLM16 | 64 | M | IV | None | Mets, Liver/lung | *0401 | NA | NA |

Abbreviations Used: C, Chemotherapy; R, Radiotherapy; S, Surgery; DC/peptide, Dendritic Cell + Synthetic Melanoma Peptide Vaccine; IFN-α, Interferon Alpha Therapy; NED, No evidence of Disease at time of Blood Draw, NA, Not Available for Evaluation. Patient SLM5 genotyped as HLA-DR4 of indeterminate subtype. [†]Patient with ocular melanoma.

twice with HBSS to remove residual platelets and ficoll-hypaque. Cells were frozen in 90% FCS containing 10% DMSO (Sigma Chemical Co., St Louis, MO) at $10^7$ lymphocytes/vial using controlled-rate freezing technique. On the day of ELISPOT assay, cells were thawed and washed twice with HBSS. CD4+ T cells were first isolated using MACS™ (Miltenyi) anti-human CD4 beads and MiniMACS™ columns per the manufacturer's protocol. CD4+ T cell yields were typically 25–35% of starting PBMC numbers loaded, with purity exceeding 97% as assessed by flow cytometry.

### Induction of antitumour T effector lymphocytes

Autologous dendritic cells ($10^7$) were prepared as previously described by 7-day culture of plastic-adherent PBMC in GM-CSF and IL-4 (Tueting et al, 1998). Harvested, non-adherent DC were then loaded with antigen in one of three ways. DC were either: (1) pulsed with 10 uM synthetic peptides for 3–4 h at 37˚C; (2) pulsed with 250 μg/ml Mel 526 (gp100/pmel17+ tyrosinase+) lysate for 24 h at 37˚C; or (3) infected with recombinant vaccinia virus encoding the gp100/pmel17 or tyrosinase antigens (Kittlesen et al, 1998) at an MOI of 50 for 24 h at 37˚C. The resulting 'antigen-loaded' DC were washed, irradiated (3000 rad) and used to stimulate purified CD4+ T cells at a 50:1 responder-to-stimulator ratio. Primary in vitro cultures were performed in AIM-V medium containing 5% HuAB serum and 1 ng/ml of both rhIL-1 and rhIL-7 (Genzyme). Cultures were restimulated with the residual 1/2 of the DC-tumour stimulator preparation (cryopreserved for 1 week) on day 7 in AIM-V medium containing 5% HuAB serum and 10 IU/ml rhIL-2 (kind gift of Chiron Corporation, Emeryville, CA). These stimulated T cells were harvested on day 14–17 and analyzed for peptide/tumour specificity in ELISPOT assays.

### IFN-γ ELISPOT and capture of peptide-reactive CD4+ T cells

ELISPOT was performed essentially as previously described (Herr et al, 1998). Spots were imaged using the Zeiss AutoImager and statistical comparisons determined using a Student's two-tailed *t*-test analysis. The data are represented as IFN-γ spots per 100 000 CD4+ T cells analysed. HLA-restriction of CD4+ T cell responses was demonstrated by addition of blocking mAb (5 ug/well) directed against HLA-DR4 (clone 359-13F10, IgG, kindly provided by Dr Janice Blum, Indiana University School of Medicine, Indianapolis, IN).

### PCR analysis

PCR analyses were performed to determine patient HLA-DR4 genotype using a commercial PCR panel according to the manufacturer's instructions (Dynal, Oslo, Norway). RT-PCR analysis was also used to determine tumor expression of gp100/pmel17 and tyrosinase mRNA (Storkus and Zarour, 2000).

### RESULTS

### Selection and screening of candidate DR4-binding peptides derived from gp100/pmel17 and tyrosinase

In order to identify a series of candidate peptides capable of being recognized by CD4+ T cells in HLA-DRA+ patients who have, or have had melanoma, we subjected the cDNA sequences of gp100/pmel17 and tyrosinase to analysis using a computer algorithm designed to predict HLA-DR*0401-binding peptides (Honeyman et al, 1998). Nine amino acid-long 'core' sequences were evaluated and scored from 0–10, with the highest scoring sequences taken to represent peptides most likely to bind to HLA-DR*0401. A total of 18 peptides were produced (Table 1). One gp100/pmel17 (gp100/pmel17$_{44–59}$) and two tyrosinase peptides (tyrosinase$_{56–70}$ and tyrosinase$_{448–462}$), previously identified to be recognized by DR4-restricted CD4+ TIL (Storkus and Zarour, 2000), were included as 'positive controls' among the panel used in this study. Based on the predictive algorithm used, these 'control' peptides would have been produced for analysis due to their inclusion of core epitopes yielding algorithm scores ≥ 4 (Table 1).

 *© 2001 Cancer Research Campaign*

These selected synthetic peptides were then assessed for their comparative abilities to bind to purified HLA-DR4 molecules using a solid-state competitive-binding assay (Southwood et al, 1998). The data reported as the dose of peptide capable of inhibiting 50% of the binding of a reference DR4-binding peptide ($IC_{50}$ in nM) are listed in Table 1. Most of the peptides evaluated bound both HLA-DR4 alleles, albeit over a wide range of observed $IC_{50}$ values. The strongest HLA-DR4 (both HLA-DRB1*0401 and -DRB1*0404) binding peptides for each antigen tested were $gp100_{615-633}$ (identical to $pmel17_{622-640}$) and $tyrosinase_{365-381D}$, respectively (Table 1).

### Immunoreactivity of CD4+ T cells against predicted HLA-DR4-binding tumour peptides in HLA-DR4+ melanoma patients

In a preliminary screen of the immunogenicity of these peptides, we evaluated the ability of CD4+ T cells isolated directly from the peripheral blood of 16 HLA-DR4+ patients treated for melanoma (Table 2) to recognize these putative peptide epitopes using the IFN-γ ELISPOT assay. Eleven of these individuals (SLM1-SLM11) were rendered disease-free as a result of surgery and/or immunotherapy and we hypothesized that their lack of current disease might, in part, be attributed to circulating anti-melanoma CD4+ T cells. As indicated in Tables 1 and 3 and Figure 1A, a number of these disease-free patients displayed detectable frequencies of circulating Th0/Th1-type (i.e. IFN-γ secretors) CD4+ T cells that recognized a sub-set of the peptides selected for analysis (i.e. gp100/$pmel17_{6-26}$, gp100/$pmel17_{44-59}$, gp $100_{615-633}$/$pmel17_{622-640}$, $tyr._{56-70}$, $tyr._{156-175}$, $tyr._{365-381D}$ and $tyr._{365-381N}$). Interestingly, with the exception of a single HLA-DR4+ patient with ocular melanoma (SLM12), four additional patients (SLM13-SLM16) with active stage III or IV disease exhibited minimal or no reactivity to any of the peptides analysed in IFN-γ ELISPOT assays (Figure 1B). These same peptides failed to be recognized, or were recognized extremely poorly by freshly-isolated CD4+ T cells harvested from a series of 10 normal HLA-DR4+ donors (Figure 1C).

### Immunoreactivity of CD4+ T cells against predicted HLA-DR4-binding tumour peptides in HLA-DR4+ normal donors after in vitro stimulation

Plastic non-adherent PBMC derived from the HLA-DR4+ normal donors were stimulated with autologous DC that had been pre-pulsed with a given gp100/pmel17 or tyrosinase peptide identified in Table 1. One week after restimulation of T cells with identically prepared autologous DC, CD4+ T cells were purified by immunomagnetic procedures and used as responders against T2.DR4 target cells pulsed with the candidate DR4-binding peptides and against an HLA-DR4+/gp100+/ tyrosinase+ melanoma cell line SLM2 in IFN-γ ELISPOT assays. Cryopreserved, freshly-isolated (i.e. non-stimulated) CD+ T cells obtained from each donor were also analysed in these same assays in order to determine the basal ‘in situ’ level and the impact of in vitro stimulation on the calculated frequencies of peptide-specific responder CD4+ T cells in these individuals. As shown in Tables 1 and 3 and Figure 2, high-frequency responses (as high as 1/119 against the $gp100_{615-633}$ (identical to $pmel17_{622-640}$ peptide)) were observed from in vitro stimulated CD4+ T cells against 3/11 gp100/pmel17-and 4/7 tyrosinase-derived peptides using the IFN-γ ELISPOT assay. On an arbitrary scale, the gp100/$pmel17_{6-26}$ served as a ‘weak’ immunogen, the $tyrosinase_{156-175}$, $tyrosinase_{178-194}$, and $tyrosinase_{365-381N}$ served as ‘moderate’ immunogens, and the gp100/$pmel17_{44-59}$, $gp100_{615-633}$ (identical to $pmel17_{622-640}$), $tyrosinase_{56-70}$ and $tyrosinase_{365-381D}$ served as strong immunogens in both melanoma patients and (in vitro stimulated) normal donors (Table 1). Two of these peptides represent previously-defined ‘helper’ T cell epitopes in the melanoma setting ($tyrosinase_{56-70}$, gp100/$pmel17_{44-59}$, Storkus and Zarour, 2000; Touloukian et al, 2000). The previously defined

**Table 3** Significance of differences in epitope reactivity between donor groups evaluated

| Peptide evaluated | *P* values for differences between groups | | |
|---|---|---|---|
| | Patients NED vs patients with disease | Patients NED vs normal donors | Patients with disease vs normal donors |
| gp 100/$pmel17_{6-26}$ | 0.325 | <u>0.002</u> | <u>0.031</u> |
| gp 100/$pmel17_{44-59}$** | <u>0.036</u> | <u>0.003</u> | 0.455 |
| gp 100/$pmel17_{150-169}$ | 0.070 | 0.152 | 0.353 |
| gp 100/$pmel17_{167-189}$ | 0.174 | 0.532 | 0.353 |
| gp 100/$pmel17_{283-304}$ | 0.178 | 0.372 | 0.352 |
| gp 100/$pmel17_{377-398}$ | 0.377 | 0.480 | 0.155 |
| gp 100/$pmel17_{400-421}$ | 0.546 | 0.234 | 0.234 |
| gp 100/$pmel17_{506-522}$ | 0.546 | 0.890 | 0.527 |
| gp 100/$pmel17_{540-561}$ | 0.376 | 0.685 | 0.527 |
| gp $100_{599-619}$/$pmel17_{606-626}$ | 0.178 | 0.155 | 0.527 |
| gp $100_{615-633}$/$pmel17_{622-640}$ | <u>0.043</u> | <u>0.002</u> | 0.184 |
| $tyrosinase_{56-70}$** | <u>0.026</u> | <u>0.001</u> | 0.187 |
| $tyrosinase_{80-95}$ | 0.376 | 0.602 | 0.527 |
| $tyrosinase_{156-175}$ | <u>0.010</u> | <u>0.001</u> | 0.485 |
| $tyrosinase_{178-194}$ | 0.618 | 0.074 | 0.693 |
| $tyrosinase_{365-381D}$ | 0.132 | <u>0.005</u> | 0.606 |
| $tyrosinase_{365-381N}$ | 0.687 | <u>0.003</u> | 0.077 |
| $tyrosinase_{448-462}$** | 0.262 | 0.303 | 0.527 |

Two-tailed significance of Figure 1 data analyzed by Mann-Whitney (non-parametric) test.
*P* values < 0.05 are underlined.

*© 2001 Cancer Research Campaign*

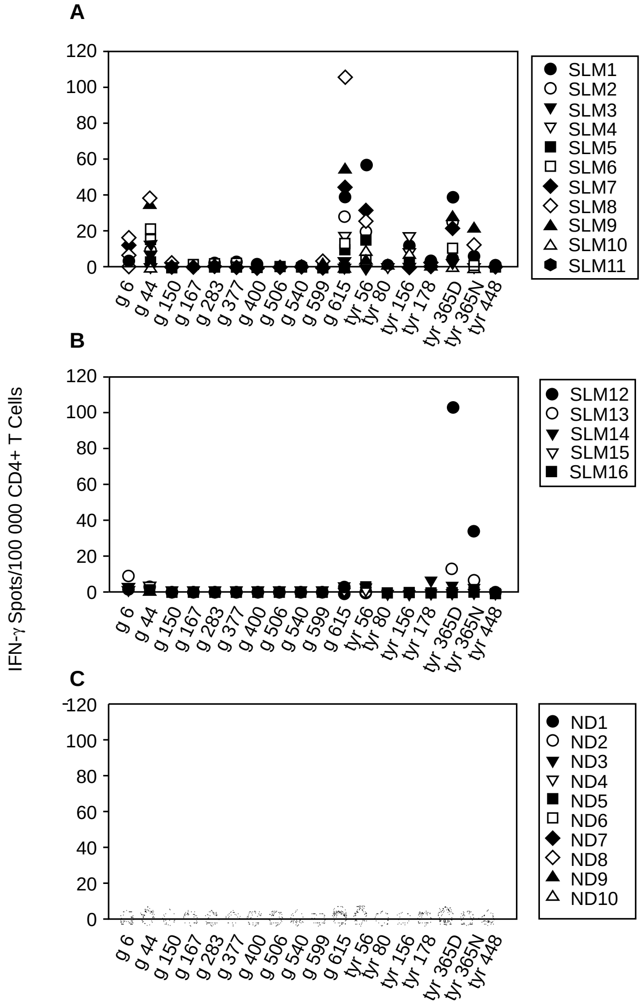

**Figure 1** Freshly-prepared peripheral blood CD4+ T cell reactivity of HLA-DR4+ patients with melanoma against gp100/pmel17-and tyrosinase-derived peptides. Peripheral blood CD4+ T cells were freshly-prepared from HLA-DR4+ melanoma patients (see Table 2) and normal donors and analyzed for their reactivity to the algorithm-selected gp 100/pmel17 and tyrosinase peptides using 20 h IFN-γ ELISPOT assays. Depicted are the results from 11 melanoma patients currently free of disease (panel A), 5 melanoma patients with active disease (panel B) and 10 normal donors (panel C). Data depicted represent the mean of triplicate determinations from which the mean of CD4+ T cell responses against the control T2.DR4 (i.e. no peptide) presenting cell line has been subtracted (in all cases, the mean of this background control value was ≤ 6 spots). To determine positive responsiveness as summarized in Table 1, patient mean data (spots per 100,000 CD4+ T cells) for a given peptide had to exceed the mean + 3 S.D. of the corresponding spot number obtained from the 10 normal donors. The normal mean + 3 S.D. values for the individual peptides were: $gp100_{6–26}$ (1.4); $gp100_{44–59}$ (3.7); $gp100_{150–169}$ (1.8); $gp100_{167–179}$ (1.8); $gp100_{283–304}$ (1.4); $gp100_{377–398}$ (1.5); $gp100_{400–421}$ (1.6); $gp100_{506–522}$ (1.5); $gp100_{540–561}$ (1.4); $gp100_{599–619}$ (1.4); $gp100_{615–633}$ (3.9); $tyr._{56–70}$ (4.9); $tyr._{80–95}$ (1.0); $tyr._{156–175}$ (1.0); $tyr._{178–194}$ (1.3); $tyr._{365–381D}$ (5.3); $tyr._{365–381N}$ (1.3); $tyr._{448–462}$ (1.0)

$tyrosinase_{448–462}$ epitope (Topalain et al, 1996), despite binding to HLA-DRB1*0401 with moderate ‘affinity’ (Table 1), failed to be recognized by CD4+ T cells derived from either HLA-DRB1*0401+ patients or normal donors (Table 1, Figures 1 and 2). As depicted in Figure 2, CD4+ T cells recognizing specific peptides also recognized the SLM2 melanoma cell line (columns B and D in each panel) and this recognition was inhibited by inclusion of anti-DR4 mAb in the assay (columns C and F in each panel).

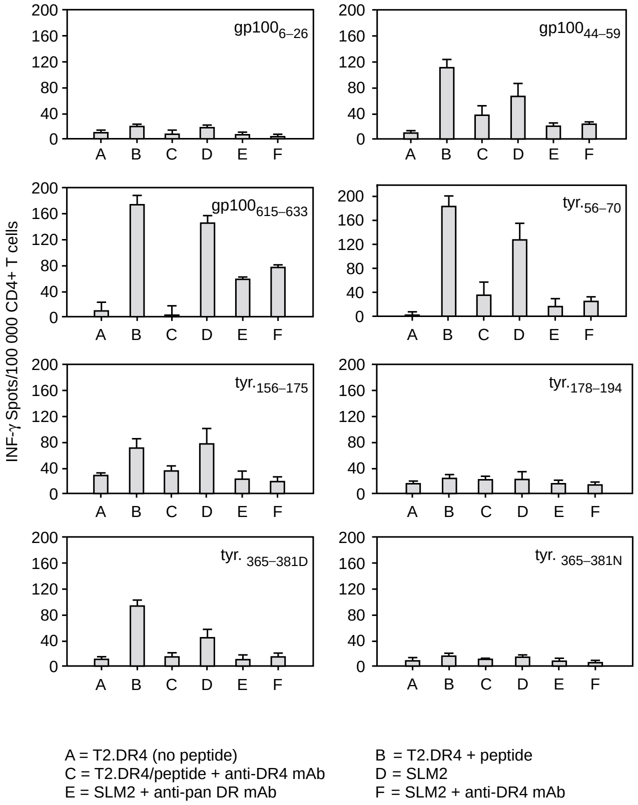

**Figure 2** Reactivity of HLA-DR4+ normal donor CD4+ T cells after in vitro sensitization with gp100/pmel17-and tyrosinase-derived peptides. HLA-DR4+ normal donor peripheral blood CD4+ T cells were stimulated with autologous monocyte-derived DC pre-pulsed with the indicated gp100/pmel17 and tyrosinase peptides. After an additional restimulation with identically prepared DC one week later (i.e. day 7 of culture), the resultant purified CD4+ T cell populations were analyzed for peptide-specific reactivity in 20 h IFN-γ ELISPOT assays (i.e. on day 14–17 of culture). Individual target groups are indicated. Data are representative for 1 of 10 normal donors evaluated and are qualitatively summarized in Table 1

### CD4+ T cells stimulated in vitro with autologous DC infected with vaccinia encoding gp100/pmel17 or tyrosinase or pulsed with melanoma lysates recognize DR4-presented melanoma peptides

We next investigated whether autologous HLA-DR4+ DC that have acquired gp100/pmel17 or tyrosinase as a result of infection with a recombinant vaccinia virus or via exogenous loading with a freeze-thaw lysate derived from the allogeneic DR4-negative, gp100/pmel17+, tyrosinase+ Mel 526 promoted the activation and expansion of epitope-specific normal donor CD4+ T cells in vitro. In VAC-gp100/DC stimulated cultures, CD4+ T cells preferentially recognized peptides $gp100_{44–59}$ and $gp100_{615–633}$ (identical to $pmel17_{622–640}$), and reacted against 2/4 of the HLA-DR4+/gp100+ melanoma cell lines analyzed (Figure 3). CD4+ T cell cultures stimulated with autologous VAC-tyrosinase infected DC recognized 4 of 6 peptides and 2 of 4 melanoma cell lines evaluated (Figure 3). CD4+ T cells stimulated with autologous HLA-DR4+ DC pre-pulsed with a lysate derived from the Mel 526 cell line were cytolytic when tested against HLA-DR4+, gp100/pmel17+, tyrosinase+ melanoma COLO38 (data not shown). Specific lysis of COLO38 mediated by this polyspecific T cell population could be inhibited partially by inclusion of non-radiolabeled T2.DR4 pre-pulsed with certain peptide epitopes derived from

© 2001 Cancer Research Campaign

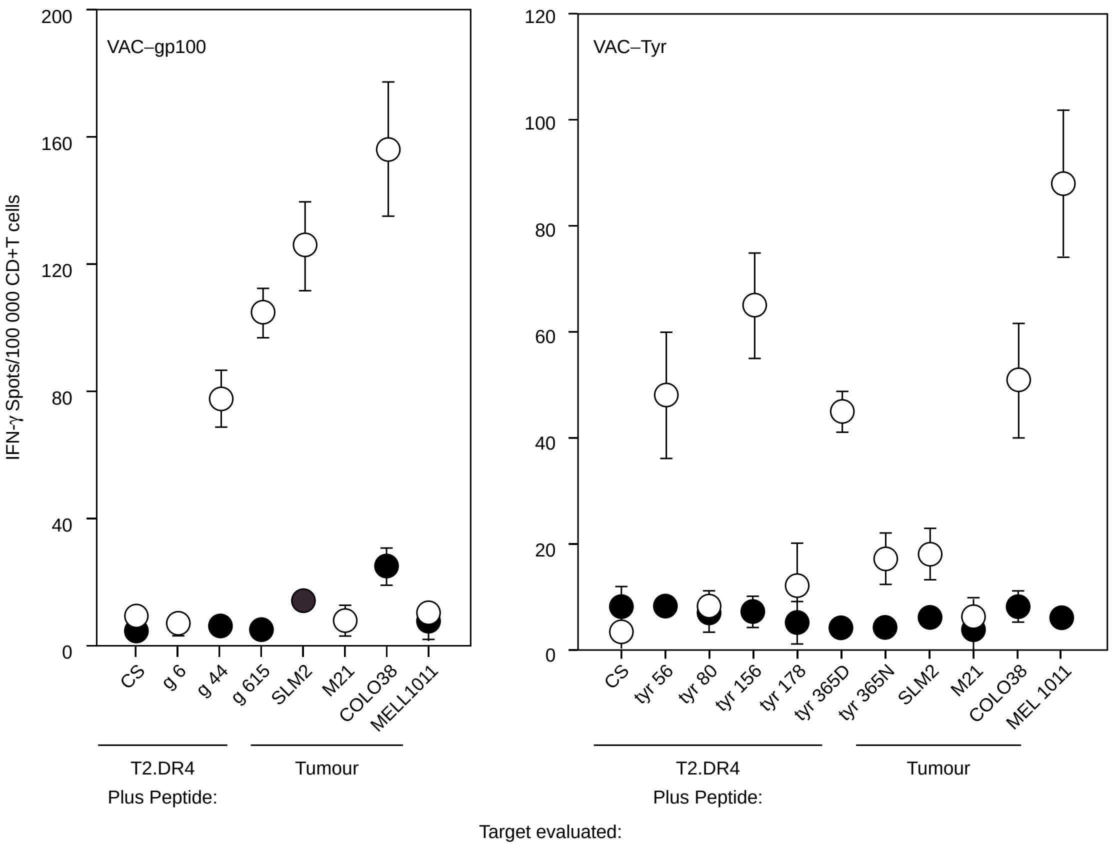

**Figure 3** DC infected with rVac-gp100 (VAC-gp100) or rVac-tyrosinase (VAC-Tyr.) elicit peptide-specific CD4+ T cells in vitro. Immature DC were generated in vitro from a normal HLA-DR4+ (DRB1*0401 +) donor and infected with the indicated recombinant vaccinia viruses at an MOI of 50 as previously described (18) and then used to stimulate autologous CD4+ T cells at a responder-to-stimulator ratio of 20:1. After being restimulated with identically prepared DC on day 7 of culture, CD4+ T cells were harvested and evaluated for their reactivity against peptide-pulsed T2.DR4 target cells or a series of melanoma cell lines in 20 h IFN-γ ELISPOT assays one week later (open symbol). The mean of background T cell responses against the T2.DR4 target (without peptide) have been subtracted in all cases of peptide testing, but were 23 spots and 16 spots per 100,000 CD4+ T cells in the Vacc-gp100 and Vacc-tyrosinase cultures, respectively. T cells responses to melanoma target cell lines were not corrected and represent actual spot numbers. Peptide CS is an HLA-DR4-presented epitope derived from the malarial circumsporozooite protein that serves as a negative biological control. Melanoma cell line phenotypes are as follows: SLM2 is HLA-DR4+, gp100/pmel17+, tyrosinase+; M21 is HLA-DRdim+, gp100/pmel17+, tyrosinase+; COLO38 is HLA-DR4+, gp100/pmel17+, tyrosinase+; and Mel1011 is HLA-DR4+, gp100/pmel17+/–, tyrosinase+ (see Materials and Methods). The DR4-restricted nature of T cell reactivity was demonstrated by addition of blocking anti-HLA-DR4 mAb 359-13F10 (filled symbol). Data represent the mean +/– SD of triplicate determinations from 1 of 3 donors evaluated. All donors displayed similar reactivity patterns.

gp100/pmel17 and tyrosinase, with the $gp100_{615–633}$ and $tyrosinase_{56–70}$ peptides proving to be most effective (data not shown). Overall, this suggests that immunogenic core epitope(s) resident within each of these biologically-active, synthetic sequences is naturally processed and presented by antigen-loaded host DC and by HLA-DR4+ melanomas that constitutively express the relevant antigens.

### DISCUSSION

Using HLA-DR4+ patient's PBMC, a peptide-binding algorithm, and a high-sensitivity IFN-γ ELISPOT assay, we have identified three novel, non-overlapping epitopes derived from the gp100/pmel17 and tyrosinase melanoma-associated antigens (MAA) that are functionally recognized by Th1-type CD4+ T cells. In addition, two of three previously identified CD4+ epitopes derived from gp100/pmel17 and tyrosinase ($gp100/pmel17_{44–59}$ and $tyrosinase_{56–70}$ but not $tyrosinase_{448–462}$) were also confirmed in these analyses (Topalian et al, 1996; Storkus and Zarour, 2000; Touloukian et al, 2000). Overall, 7/18 peptides chosen for evaluation (based on the algorithm analysis) appeared to promote Th1-type responses from purified CD4+ T cells obtained from melanoma patients. CD4+ T cells from normal HLA-DR4+ donors were poorly reactive against epitopes and, with the exception of the dramatic anti-tyrosinase responses noted for a single patient with ocular melanoma (i.e. SLM12), CD4+ T cells from patients with active disease also tended to display muted peptide-specific responses. In marked contrast, CD4+ T cells isolated from the PBMC of patients that have been effectively treated in the clinic and that remain disease-free, displayed significantly elevated responses to a number of these putative epitopes. This suggests that long-term 'memory' CD4+ T cells may circulate at high frequency in the periphery of these 'protected' patients.

Alternatively or additionally, the T cell repertoires of these patients may have been periodically restimulated (by clinically undetectable, recurrent micrometastatic disease) by either class II+ tumours themselves or via 'cross-presentation' by tumour-associated antigen presenting cells (Ossendorp et al, 1998; Albert et al, 1998; Lotze et al, 1997). Our data provided in Figure 3 and support the ability of CD4+ T cells to recognize processed epitopes 'cross-presented' by autologous DC. In particular, novel epitopes within

© 2001 Cancer Research Campaign 

the gp100$_{615–633}$, tyrosinase$_{156–175}$ and tyrosinase$_{365–381D/N}$ peptides appeared to be naturally presented by relevant antigen cDNA transduced DC (while those for gp100$_{6–26}$ and tyrosinase$_{178–194}$ were not). Such cognate recognition of cross-presented epitopes in situ may promote the function or durability of infiltrating CD8+ T cells by enhancing CTL cytolytic activity or increasing target cell recognition via upregulation of MHC class I molecule expression (i.e. IFN-γ) and by protecting T cells against tumour-induced apoptosis (Lorenz et al, 1997).

Of the three novel, non-overlapping epitopes defined in this study, one was derived from the gp100/pmel17 protein (gp 100$_{615–633}$ (identical to pmel17$_{622–640}$)) and two were derived from the tyrosinase protein (tyrosinase$_{156–175}$ and tyrosinase$_{365–381D}$ (overlaps with tyrosinase$_{365–381N}$)). While the gp100/pmel17$_{6–26}$ peptide was reacted against by CD4+ T cells obtained from melanoma patients free of disease and normal donors stimulated with peptides in vitro (Table 1), these responses were very modest and were not observed in cultures stimulated by Vac-gp100-infected autologous DC (Figure 3). This could reflect the differential processing of this gp100 epitope by melanomas versus transduced DC, given variance in processing observed amongst antigen-presenting cells (Vidard et al, 1992), or it could reflect T cell cross-reactivity against another naturally-processed sequence expressed in the melanoma setting. Given these uncertainties, we do not classify this peptide as containing a naturally-processed epitope at this time.

Interestingly, one of the HLA-DR4 presented tyrosinase epitopes (i.e. tyrosinase$_{365–381}$) encompasses a glycosylation site known to be post-translationally-modified in melanoma cells. Thus, the genomically-encoded tyrosinase contains an asparagine (N, single letter designation) residue at position 370 that is modified into an aspartic acid (D, single letter designation) as a result of enzymatic processing. Skipper et al (1996) have previously demonstrated that this modification yields the naturally-processed and HLA-A2-presented tyrosinase$_{368–376}$ (YMDGTMSQV) epitope. We have now similarly shown that a ‘post-translationally modified’ tyrosinase$_{365–381D}$, but not the genomically-encoded tyrosinase$_{365–381N}$ peptide, serves as a strong HLA-DR4-presented immunogen recognized by melanoma-reactive CD4+ T cells. The observed enhanced immunoreactivity of the tyrosinase$_{365–381D}$ peptide can be attributed, in part, to its approximately 100-fold better binding ‘affinity’ to HLA-DRB1*0401 (vs the tyrosinase$_{365–381N}$ peptide, Table 1). The lack of cross-reactivity of responder CD4+ T cells against these two epitopes, however, may also argue for non-overlapping peptide-specific T cell repertoires.

The immunogenic gp100/pmel17 and tyrosinase epitopes identified in this study are presented by HLA-DR4, which is expressed by 18–20% of the population afflicted with melanoma (Gjertson and Lee, 1998). The clinical utility of these sequences would be enhanced greatly if they could be presented in the context of additional HLA-DR alleles to melanoma-reactive CD4+ T cells. Our preliminary analysis suggests that a number of these epitopes bind to a broad range of MHC class II alleles (data not shown). For instance, the gp100/pmel17$_{44–59}$ peptide binds to (at least) the HLA-DR1, -DR3, -DR4, -DR7, -DR13 and -DRw53 (i.e. -DRB4*0401) alleles that cover in excess of one-half of the American population (Gjertson and Lee, 1998). If naturally presented by these non-HLA-DR4 alleles, these pan-DR binding peptides may prove immunogenic to a broad range of patients, a possibility that we are currently evaluating.

While we have identified a series of biologically-active epitopes in this study, we have little conclusive data (save for confirmation of the gp100/pmel17$_{44–59}$ sequence, recently identified as a natural epitope (Storkus and Zarour, 2000; Touloukian et al, 2001) to support that these sequences are ‘naturally-presented’ in the exact format as tested. We are currently performing mass spectrometric analyses of peptides derived from affinity-purified HLA-DR4 complexes obtained from MHC class II+ melanoma cell lines in order to determine a series of natural tumour-processed ‘minimal core epitopes’. From a practical perspective however, the identification of a ‘minimal core epitope’ may be a subordinate issue in the context of the potential clinical applications of these peptides, which include immune monitoring and vaccine construction. Long peptides (i.e. 12–16 amino acids) containing a core 9-mer epitope have been previously shown to elicit protective immunity mediated by CD8+ T cells in the in vivo vaccine setting (Kast et al, 1993) or in vitro after direct binding of peptide to the MHC molecule (Kittlesen et al, 1998 and unpublished observations). Similar mechanisms would likely allow for ‘optimal’ loading of HLA-DR complexes with relevant epitopes that are typically not as constrained for length as are MHC class I-presented peptides (Falk et al, 1994). This might be best analysed in preclinical in vivo systems such as the DR-IE transgenic mouse and SCID-Hu vaccine models that have been recently used to corroborate or define the gp100$_{44–59}$ or gp100$_{231–243}$ epitopes (Touloukian et al, 2000; Cochlovius et al, 2000). The latter model (Cochlovius et al, 2000) in particular, could provide strong support for the clinical utility of vaccines incorporating HLA-DR presented epitopes since DC-peptide vaccines may inhibit the growth of melanoma cell lines expressing the relevant antigen (i.e., from which the peptide derived) in situ.

A potentially added benefit to the clinical use of certain of the identified gp100/pmel17 and tyrosinase peptides is that they contain embedded CTL epitopes (Table 1). Hence, a single synthetic peptide may yield both CD4+ and CD8+ T cell recognized determinants when appropriately processed in situ, by at least some patients (bearing the appropriate MHC class I alleles for CTL epitope presentation). Such combination vaccines containing ‘helper’ T and CTL epitopes may prove particularly therapeutic (Ranieri et al, 2000).

### ACKNOWLEDGEMENTS

The authors wish to thank Mr William Knapp for his excellent technical support and to Drs Wolfgang Herr and Jan Mueller-Berghaus, and Mrs Anna Kalinska for their careful review and comments during the generation of this manuscript. This work was supported by National Institutes of Health (NIH) grants CA57840 (WJS) and CA57653 (CLS), and NIH contract N01-AI 95362 (AS).

### REFERENCES

Albert ML, Sauter B, Bhardwaj N (1998) Dendritic cells acquire antigen from apoptotic cells and induce class I-restricted CTLs. *Nature* **392**: 86–89

Aruga A, Aruga E, Cameron MJ and Chang AE (1997) Different cytokine profiles released by CD4+ and CD8+ tumor-draining lymph node cells involved in mediating tumor regression. *J Leuk Biol* **61**: 507–516

Barger BO, Acton RT, Soong SJ, Roseman J and Balch C (1982) Increase of HLA-DR4 in melanoma patients from Alabama. *Cancer Res* **42**: 4276–4279

Bour H, Horvath C, Lurquin C, Cerottini JC and MacDonald HR (1998) Differential requirement for CD4 help in the development of an antigen-specific CD8+ T cell response depending on the route of immunization. *J Immunol* **160**: 5522–5529

Brinckerhoff LH, Thompson LW and Slingluff CL Jr (2000) Melanoma vaccines. *Curr Opin Oncol* **12**: 163–173

 *© 2001 Cancer Research Campaign*

Cochlovius B, Stassar M, Christ O, Raddrizzani L, Hammer J, Mytilineos I and Zoller M (2000) In vitro and in vivo induction of a Th cell response toward peptides of the melanoma-associated glycoprotein 100 protein selected by the TEPITOPE program. *J Immunol* **165**: 4731–4741

Czarnecki D, Nicholson I, Tait B and Nash C (1993) HLA DR4 is associated with the development of multiple basal cell carcinomas and malignant melanoma. *Dermatol* **187**: 16–18

Falk K, Rotzschke O, Stevanovic S, Jung G and Rammensee HG (1994) Pool sequencing of natural HLA-DR, DQ, and DP ligands reveals detailed peptide motifs, constraints of processing, and general rules. *Immunogenetics* **39**: 230–242

Gjertson DW and Lee SH (1998) *HLA 1998*. Gjertson DW and Terasaki PI (eds) p 450. Lenexa, KS: Am. Soc. Histocompatibility and Immunogenetics

Grohmann U, Fioretti MC, Bianchi R, Belladonna ML, Ayroldi E, Surace D, Silla S and Puccetti P (1998) Dendritic cells, interleukin 12, and CD4+ T lymphocytes in the initiation of class I-restricted reactivity to tumor/self peptide. *Crit Rev Immunol* **18**: 87–98

Halliday GM, Patel A, Hunt MJ, Tefany FJ and Barnetson RS (1995) Spontaneous regression of human melanoma/nonmelanoma skin cancer: association with infiltrating CD4+ T cells. *World J Surg* **19**: 352–358

Herr W, Protzer U, Lohse AW, Gerken G, Meyer zum Buschenfelde KH and Wolfel T (1998) Quantification of CD8+ T lymphocytes responsive to human immunodeficiency virus (HIV) peptide antigens in HIV-infected patients and seronegative persons at high risk for recent HIV exposure. *J Inf Dis* **178**: 260–265

Honeyman MC, Brusic V, Stone NL and Harrison LC (1998) Neural network-based prediction of candidate T-cell epitopes. *Nature Biotechnol* **16**: 966–969

Kast WM, Brandt RM and Melief CJ (1993) Strict peptide length is not required for the induction of cytotoxic T lymphocyte-mediated antiviral protection by peptide vaccination. *Eur J Immunol* **23**: 1189–1192

Kawakami Y, Robbins PF, Wang RF, Parkhurst M, Kang X and Rosenberg SA (1998) The use of melanosomal proteins in the immunotherapy of melanoma. *J Immunother* **21**: 237–246

Kittlesen DJ, Thompson LW, Gulden PH, Skipper JC, Colella TA, Shabanowitz J, Hunt DF, Engelhard VH, Slingluff CLJr and Shabanowitz JA (1998) Human melanoma patients recognize an HLA-A1-restricted CTL epitope from tyrosinase containing two cysteine residues: implications for tumor vaccine development. *J Immunol* **160**: 2099–2106

Lorenz HM, Hieronymus T, Grunke M, Manger B and Kalden JR (1997) Differential role for IL-2 and IL-15 in the inhibition of apoptosis in short-term activated human lymphocytes. *Scand J Immunol* **45**: 660–669

Lotze MT, Hellerstedt B, Stolinski L, Tueting T, Wilson C, Kinzler D, Vu H, Rubin JT, Storkus WJ, Tahara H, Elder E and Whiteside T (1997) The role of interleukin-2, interleukin-12, and dendritic cells in cancer therapy. *Cancer J Sci Am* **3S**: 109–114

Matloubian M, Concepcion RJ and Ahmed R (1994) CD4+ T cells are required to sustain CD8+ T-cell responses during chronic viral infection. *J Virol* **68**: 8056–8063

Ogoshi K, Tajima T, Mitomi T and Tsuji K (1994) HLA-DR4 antigen and lymph node metastases in poorly differentiated adenocarcinoma of the stomach. *Cancer* **73**: 2250–2252

Ossendorp F, Mengede E, Camps M, Filius R and Melief CJ (1998) Specific T helper cell requirement for optimal induction of cytotoxic T lymphocytes against major histocompatibility complex class II negative tumors. *J Exp Med* **187**: 693–702

Ranieri E, Kierstead LS, Zarour H, Kirkwood JM, Lotze MT, Whiteside T and Storkus WJ (2000) Dendritic cell/peptide cancer vaccines: clinical responsiveness and epitope spreading. *Immunol Inv* **29**: 121–125

Sgadari C, Angiolillo AL, Cherney BW, Pike SE, Farber JM, Koniaris LG, Vanguri P, Burd PR, Sheikh N, Gupta G, Teruya-Feldstein J and Tosato G (1996) Interferon-inducible protein-10 identified as a mediator of tumor necrosis in vivo. *Proc Natl Acad Sci USA* **93**: 13791–13796

Skipper JC, Hendrickson RC, Gulden PH, Brichard V, Van Pel A, Chen Y, Shabinowitz J, Wolfel T, Slingluff CL Jr., Boon T, Hunt DF and Engelhard VH (1996) An HLA-A2-restricted tyrosinase antigen on melanoma cells results from posttranslational modification and suggests a novel pathway for processing of membrane proteins. *J Exp Med* **183**: 527–534

Southwood S, Sidney J, Kondo A, del Guercio MF, Appella E, Hoffman S, Kubo RT, Chesnut RW, Grey HM and Sette A (1998) Several common HLA-DR types share largely overlapping peptide binding repertoires. *J Immunol* **160**: 3363–3373

Storkus WJ and Zarour HM (2000) Melanoma antigens recognised by CD8+ and CD4+ T cells. *Forum (Genova)* **10**: 256–270

Tannebaum CS, Tubbs R, Armstrong D, Finke JH, Bukowski RM and Hamilton TA (1998) The CXC chemokines IP-10 and Mig are necessary for IL-12-mediated regression of the mouse RENCA tumor. *J Immunol* **161**: 927–932

Topalian SL, Gonzales MI, Parkhurst M, Li YF, Southwood S, Sette A, Rosenberg SA and Robbins PF (1996) Melanoma-specific CD4+ T cells recognize nonmuted HLA-DR-restricted tyrosinase epitopes. *J Exp Med* **183**: 1965–1971

Touloukian CE, Leitner WW, Topalian SL, Li YF, Robbins PF, Rosenberg SA and Restifo NP (2000) Identification of a MHC class II-restricted human gp 100 epitope using DR4-IE transgenic mice. *J Immunol* **164**: 3535–3542

Tueting T, Wilson CC, Martin DM, Kazamon Y, Rowles J, Ma DI, Slingluff CLJr, Wagner SN, van der Bruggen P, Baar J, Lotze MT and Storkus WJ (1998) Autologous human monocyte-derived dendritic cells genetically modified to express melanoma antigens elicit primary cytotoxic T cell responses *in vitro*: Enhancement by cotransfection of genes encoding the Th1-biasing cytokines IL-12 and IFN-α. *J Immunol* **160**: 1139–1147

Tuvy DN and Blum JS (1998) Detection of biotinylated cell surface receptors and MHC molecules in a capture ELISA: a rapid assay to measure endocytosis. *J Immunol Meth* **212**: 9–18

Vidard L, Rock KL and Benacerraf B (1992) Heterogeneity in antigen processing by different types of antigen-presenting cells. Effect of cell culture on antigen processing ability. *J Immunol* **149**: 1905–1911

*© 2001 Cancer Research Campaign*
